# Diabetes and climate change: breaking the vicious cycle

**DOI:** 10.1186/s12916-023-02980-x

**Published:** 2023-07-31

**Authors:** B.M.C. Medicine

**Affiliations:** The Campus, 4 Crinan Street, London, UK

Climate change is one of the biggest and most devastating challenges facing this world with climate action claiming a place as the SDG13 of the United Nations. The vicious cycle consisting of human decisions influencing weather changes and the consequences of such changes has created a dilemma that is quite perplexing to solve. The rise in global temperatures has led to many drastic impacts worldwide, one of which is on health. Some of the most vulnerable victims of climate change are those living with diabetes. At first glance, one may wonder what the association between the two is, but a closer look reveals an underlying bidirectional relationship hidden in the fabric of this problem. With both climate change and diabetes rates worsening year on year, this editorial will turn its attention to the impact of climate change on diabetes and vice versa, as well as the constructive moves that could help to mitigate the damages caused by both problems.

Specific characteristics of climate change such as heat waves, air pollution, and extreme weather events can have severe consequences for vulnerable individuals with diabetes. The rise in ambient temperatures can increase the risk of dysregulated blood glucose levels for those using blood glucose-lowering medication. This may put patients with diabetes, particularly those above 65 years of age and with cardiovascular comorbidities, at higher risk of needing medical attention. Air pollution is also linked to an increase in risk of insulin resistance and development of diabetes as well as complications from diabetes. Nearly one-fifth of the global burden of type 2 diabetes (T2D) has been estimated to be potentially attributed to air pollution due to presence of fine particulate matter (PM 2.5) which is also a major air pollutant in wildfire smoke, another consequence of climate change. In fact, other natural disasters resulting from climate change e.g., extreme weather conditions can pose numerous challenges such as reduced access to primary care, hospitals, and medication in addition to high levels of psychological stress and lifestyle changes, which can influence glycaemic control and the management of diabetes. Lastly, another significant issue that affects individuals with diabetes as a result of climate change is transmission of infectious diseases. More than 58% of viral, bacterial, and fungal infections have been found to be worsened by climate change. This is particularly worrying since people with diabetes are known to have a higher risk of severe infections and hospitalization for bacterial and viral diseases partly due to altered immune responses.

On the flip side, let’s consider the impact of healthcare and diabetes management on the environment. Nearly 5% of the world’s greenhouse gas emissions are associated with the healthcare industry. The management of diabetes and its complications accounts for a substantial proportion of costs in any health service and produces large quantities of carbon emissions. For instance, single-use plastic is an important component of insulin pens, continuous glucose sensors, test strips, and lancets. Although plastic forms a significant portion of insulin pens (nearly 77%), it cannot be discarded in recycling bins along with other recyclable house waste material such as food packaging and therefore end up in landfills.

As well as medical management of diabetes, lifestyle factors linked to increased risk of developing T2D are themselves associated with climate change-related factors. For example, our increasing consumption of red meat is linked to greater production of greenhouse gasses and the worsening of climate change as well as an elevated risk of developing T2D. In addition, obesity, which is a factor in elevating diabetes risk, increases dietary energy requirement by approximately 19% compared to that needed by non-obese individuals. On a positive note, well-managed cases of T2D have been found to have a 7% lower carbon emission than poorly managed cases. Therefore, adopting more environmentally friendly lifestyles and sustainable mindsets is more critical than ever.

There are steps that can be taken by patients, pharmaceutical companies, and health care systems to diminish the environmental impact of diabetes. For example, individuals could move towards more plant-based diets and reduce meat and dairy consumption or if possible, walk, and cycle more rather than use vehicles. Other simple yet effective steps include growing and maintaining plants at home, using greener ways to heat homes, and promoting sustainable and healthier lifestyles in others. Patients with diabetes could be provided with reusable insulin pens that require less packaging and storage space and generate less plastic waste as well as reduced carbon footprint. With appropriate care, these easy-to-carry reusable insulin pens can last for several years as opposed to the prefilled pens which are single use. Manufacturers have also pivoted towards tackling the issue of climate change by taking the initiative to use less plastic packaging for their products and introducing recycling programs. Such programmes allow for empty prefilled insulin pens to be recycled via local pharmacies, pre-paid Royal Mail post boxes, and home-collection service. Finally, healthcare systems have the power to amplify the magnitude of the impact from these moves on the environment, for example by working with the patients and the companies to gradually implement such changes into the diabetes management system. In fact, this process has already begun as in 2020, NHS England became the first healthcare system in the world to commit itself to reducing its carbon footprint and set the goal of becoming net zero by 2040, thus paving the way for other healthcare systems to follow the same example. To achieve this the UK government is investing over £280 million into decarbonising the NHS estate in England through the Public Sector Decarbonisation Scheme.

Climate change is linked to development of diabetes and its sequelae, but human decisions are also responsible for the impact on climate. As countries continue to keep the issue of climate change on top of their agendas, we must remember that achieving an environmentally friendly and sustainable diabetes management system is a joint effort that needs patients, manufacturers, service providers and governments to act together. As rates of obesity and diabetes are on the rise in young people, we are encouraging submissions to our Paediatric Obesity and Diabetes collection. Actions already initiated to break the cycle between diabetes, and climate change are commendable. However, persistent efforts are required to incorporate these changes on a global scale in order for the impact to be palpable in the decades to come.

